# Inhibitory Potential of Different Bilberry (*Vaccinium myrtillus* L.) Extracts on Human Salivary *α*-Amylase

**DOI:** 10.3390/molecules28155820

**Published:** 2023-08-02

**Authors:** Diana Karcheva-Bahchevanska, Mariana Nikolova, Ilia Iliev

**Affiliations:** 1 Department of Pharmacognosy and Pharmaceutical Chemistry, Faculty of Pharmacy, Medical University-Plovdiv, 4002 Plovdiv, Bulgaria; 2 Department of Biochemistry and Microbiology, Faculty of Biology, Plovdiv University “Paisii Hilendarski”, 4000 Plovdiv, Bulgaria; mariana.nikolova@uni-plovdiv.bg (M.N.); iliailiev@uni-plovdiv.bg (I.I.)

**Keywords:** *Vaccinium myrtillus* L. fruits, *α*-amylase inhibition, antioxidant activity, in vitro assays

## Abstract

Recently, consumer preferences for bilberries have increased markedly. This fact is probably related to their natural constituents, such as phenolic compounds including anthocyanins and tannins, as well as the vitamins and minerals they contain. Phenolic compounds are known for their numerous beneficial effects on human health. Moreover, bilberry fruits have been shown to inhibit the activity of carbohydrate hydrolyzing enzymes, which can significantly decrease the postprandial increase in blood glucose levels. Thus, the aim of the present study is to investigate the inhibitory effect of *Vaccinium myrtillus* L. extracts on key enzyme α-amylase, linked to type 2 diabetes. No data have been published on the inhibitory properties of *Vaccinium myrtillus* L. fruits growing wild in Bulgaria against carbohydrate enzymes. Bilberry extracts were analyzed for total polyphenols, total anthocyanin content, antioxidant activity and their inhibitory properties against *α*-amylase. The contents of flavonols, anthocyanins and stilbenes were determined by HPLC analysis. The identified flavonols in the analyzed bilberry extracts were mainly represented by quercetin derivatives as rutinoside. The predominant anthocyanins for both aqueous and organic solvents were delphinidin-3-galactoside and malvidin-3-glucoside. The results revealed that bilberry extracts are effective inhibitors of *α*-amylase, with IC_50_ values from 20.8 to 194.8 μg GAE/mL. All the samples proved to have antioxidant activity measured by three different in vitro assays (FRAP, CUPRAC and DPPH). The inhibitory properties of *V. myrtillus* L. extracts may provide a new direction in the development and research of new pharmaceuticals for the suppression of postprandial hyperglycemia in diabetic patients.

## 1. Introduction

*Vaccinium myrtillus* L. is a species belonging to the genus *Vaccinium* of the family Ericaceae [1,2,3]. The plant family Ericaceae includes more than 450 species of deciduous or evergreen shrubs, rarely small trees. In Bulgaria, in natural habitats, four species are distributed—*Vaccinium myrtillus* L. (bilberry), *Vaccinium vitis-idaea* L. (lingonberry), *Vaccinium uliginosum* L. (bog bilberry) and *Vaccinium arctostaphylos* L. (Caucasian whortleberry) [4]. The plant species *Vaccinium myrtillus* L. is a shrub reaching a height of 10–40 cm. The leaves are deciduous and the fruit is a bluish-black juicy berry with dark red flesh measuring 5–7 mm in diameter [4].

A wide array of positive health effects has been ascribed to the phytochemicals present in bilberry fruits, such as their ability to protect against cardiovascular, cancer and neurodegenerative diseases [5,6]. The consumption of these fruits is steadily increasing because of their multiple health benefits. Some of these health benefits are due to their antioxidant, antimicrobial and detoxifying effects on the body. Additionally, those high in polyphenols, such as anthocyanins, are thought to be able to initiate apoptosis in cancer cells [1]. The fruits of the studied species are known to be rich in polyphenols and other components considered to be biologically active compounds (BAC) with positive effects on various physiological processes in the human body. The phytochemicals present in bilberry fruits implicated for the above biological effects are phenolic compounds, including anthocyanins [7,8], flavonols (such as kaempferol, quercetin and myricetin), flavan-3-ols (catechins), stilbenoids and phenolic acid derivatives [9,10,11]. In the preparation of bilberry extracts, the amount of different BAC and the ratio between them depends on the type of extracting solvent and the extraction conditions. Recently, bilberry fruits were shown to inhibit the activity of carbohydrate hydrolyzing enzymes, such as *α*-amylase and *α*-glucosidase, in the prevention of type 2 diabetes [12,13]. The hydrolysis of dietary starch is the major source of glucose in the blood, with *α*-amylase and *α*-glucosidase being the key enzymes involved in starch breakdown and intestinal absorption, respectively. It is believed that the inhibition of these enzymes can significantly decrease the postprandial increase in blood glucose levels [14]. Postprandial hyperglycemia plays an important role in the development of type 2 diabetes mellitus and the chronic complications associated with the disease, such as microvascularand macrovascular disorder or neuropathy [15]. Nowadays, *α*-amylase is considered as an important therapeutic target for the management of postprandial blood glucose levels, and the inhibition of this enzyme has been the subject of research for the development of new pharmaceuticals in the treatment of diabetes [16].

The fruits of *Vaccinium myrtillus* L. have mainly been studied for their antioxidant properties, but have not been thoroughly investigated for the prevention of metabolic syndrome and type 2 diabetes in humans, diseases that have been pandemic in the last two decades.

In the present study, we investigated the influence of the component composition of the extracts obtained using different extractants and the extent of the inhibition of human *α*-amylase activity as a factor in the prevention of metabolic syndrome and type 2 diabetes in humans.

## 2. Results and Discussion

### 2.1. Total Phenolic Content (TPC)

**Figure 1:**
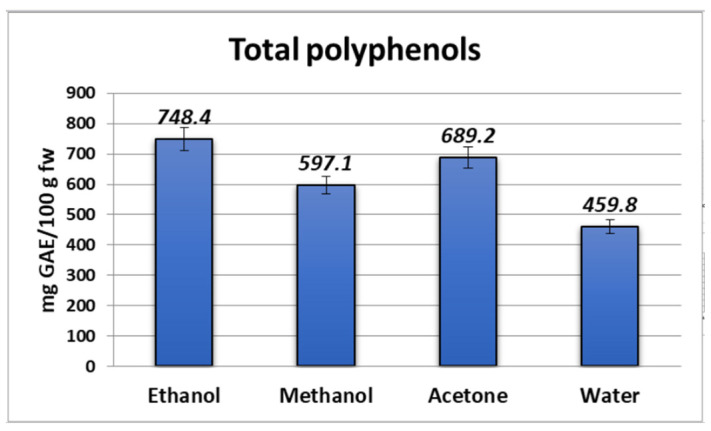
Total phenolic content in *Myrtilli fructus* extracts expressed as mg GAE/100 g fw.

The TPC ranged from 459.8 to 748.4 mg GAE/100 g fw for bilberry fruits from Bulgaria. Figure 1 shows the phenolic compound concentration in the studied bilberry extracts. The lowest amount of phenols was found in the aqueous extract, followed by the methanolic and acetone extracts by an increased amount of substances (597.1 and 689.2 mg GAE/100 g fw, respectively). The ethanolic extract of the fruits of *V. myrtillus* L. showed the highest content of total polyphenols, which was 39% more than the polyphenol content in the aqueous extraction. It can be observed that the organic samples have higher phenolic content in comparison with the aqueous one. Our data were within the range from 424.84 to 819.12 mg/100 g fw, as reported by Bunea et al. [17] and Sellappan et al. [18], which is comparable with the value reported by Prior et al. [19], which was 525 mg GAE/100 g fw for North American *V. myrtillus* L. The determined TPC values in the bilberry extracts were lower than those previously reported by Dincheva and Badjakov [20].

### 2.2. Total Anthocyanin Content (TAC)

**Figure 2:**
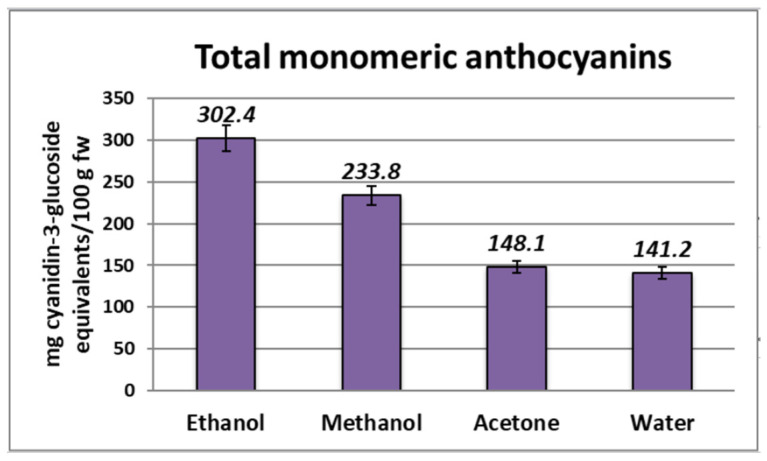
Total anthocyanin content in *Myrtilli fructus* extracts expressed as mg C3GE/100 g fw.

The TAC in the bilberry extracts from Bulgaria that were analyzed varied between 141.2 and 302.4 mg C3GE/100 g fw (Figure 2). The highest amount of anthocyanins in bilberry fruits was found for the ethanolic extract, while the lowest amount was found for the aqueous extract. A minor difference in the concentration was observed for the samples obtained using water and acetone as extraction solvents. These values were within the range reported by Bunea et al. [17]—from 69.58 to 300.02 mg/100 g which is comparable to the anthocyanin concentration found by Prior et al. [19] and higher than those reported by Sellappan et al. [18]—from 12.70 to 197.34 mg/100 g fw. Dincheva and Badjakov [20] reported even higher amounts for the studied bilberries (from 362.23 to 436.52 mgC3GE/100 g), but the TAC was determined according to Giusti and Worlstad [21].

### 2.3. Flavonol and trans-Resveratrol Content

Plant phenolic compounds such as quercetin, myricetin, anthocyanins, catechin and resveratrol were indicated to regulate glycaemia via increased glucose uptake, insulin secretion and the inhibition of lipid peroxidation, *α*-glucosidase and *α*-amylase [22,23]. 

Although different solvents are used for the extractions of phenolic compounds, rutin and myricetin were found to be the major flavonols in all the berry extracts studied. The amount of rutin and *trans*-resveratrol were determined to be almost the same in the four extracts, regardless of the extraction solvent chosen. The highest amount of myricetin was recorded in the ethanolic extract, which was 60% higher than the aqueous extract (Table 1). Flavonoids are the most common polyphenols and are widely studied as *α*-amylase inhibitors. The hydroxylation of flavonoids increases the inhibition of *α*-amylase [24]. The content of flavonols and *trans*-resveratrol in bilberry extracts is shown in Table 1. Moreover, *trans*-resveratrol was detected in bilberry samples in concentrations from 10.62 to 13.00 μg/g fw. The highest flavonol glycoside content in bilberries was represented by quercetin-rutinoside (rutin) at 110.4 μg/g fw for ethanolic extract, a value which is comparable to the one reported by Cho et al. [25].

### 2.4. Anthocyanin Content

Anthocyanins are an important class of biologically active water-soluble flavonoids. These compounds are not only associated with many positive health effects (neuroprotection, cardioprotection, etc.), but are also considered natural and safe food colorants [26]. Anthocyanins occupy a special place in the group of polyphenols found in the fruits of representatives of the genus *Vaccinium*. Their identification was performed by HPLC analysis and reported by retention times relative to a standard solution of a mixture of delphinidin-3-galactoside, delphinidin-3-glucoside, cyanidin-3-glucoside and malvidin-3-glucoside. The contents of the individual anthocyanins in the fruits of the investigated species are given in Table 2.

When using methanol as an extraction solvent, a better extraction of anthocyanins was achieved, and the amounts were consequently greater compared to aqueous extraction. However, in the bilberry extracts, the predominant glycosides for both the aqueous and organic solvent were delphinidin-3-galactoside (38.3 mg/100 g fw and 79.3 mg/100 g fw, respectively) and malvidin-3-glucoside (27.4 mg/100 g fw and 37.8 mg/100 g fw, respectively), followed by delphinidin-3-glucoside and cyanidin-3-glucoside. In agreement with the findings of other researchers, subject to similar chromatographic conditions, the order of elution of the anthocyanins is as follows: delphinidin-3-galactoside < delphinidin-3-glucoside < cyanidin-3-galactoside < delphinidin-3-arabinoside < cyanidin-3-glucoside. The last to elute are the glycosides of malvidin [10,26].

### 2.5. α-Amylase Inhibition

According to the World Health Organization (WHO), more than 400 million people are diagnosed with diabetes nowadays. Moreover, WHO predicts that in 2045 alone, around 700 million people will be affected by diabetes [16]. 

*Alpha*-amylase, which is considered one of the major enzymes in the digestion process, plays an essential role in the breakdown of polysaccharides [27]. The enzyme is mainly found in saliva and pancreatic juice [27].

Recently, *α*-amylase inhibitors of plant origin have been outlined as promising sources of novel medicines for the treatment of diabetes [27,28,29,30].

Inhibition of glucose absorption is one of the current strategies for diabetes management [31,32]. Inhibition of certain digestive enzymes responsible for the hydrolysis of polysaccharides, such as *α*-amylase, results in lower postprandial blood glucose levels [32].

Moreover, the management of postprandial hyperglycemia by regulating the activity of *α*-amylase is considered beneficial not only for controlling diabetes, but also for strategies to manage obesity and overweight [31].

**Figure 3:**
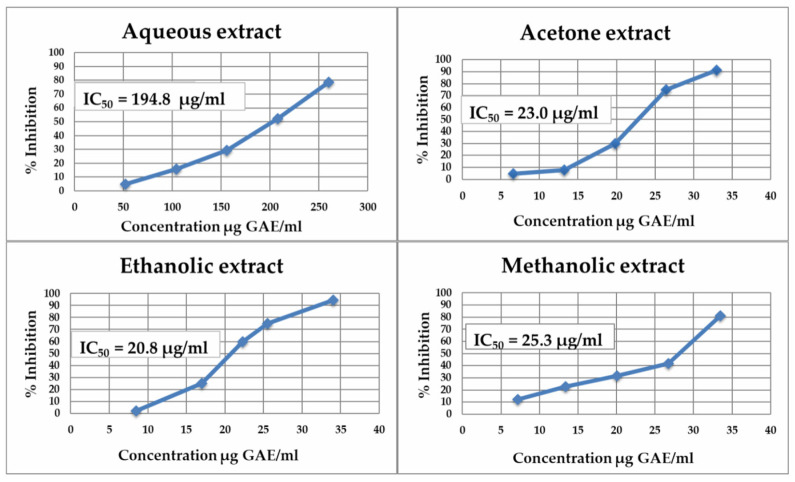
Inhibitory effect of *Myrtilli fructus* extracts on human salivary *α*-amylase activity.

The studied bilberry extracts were particularly found to be potent in terms of *α*-amylase inhibition, with IC_50_ values ranging from 20.8 to 194.8 μg GAE/mL (Figure 3). In comparison with acarbose (pseudotetrasaccharide, a natural microbial product derived from the cultures of *Actinoplanes* sp. strain SE 50), 50% inhibition has been achieved at a concentration of 5–50 µg/mL [33]. Polyphenolic organic extracts showed 4 to 5 levels more effective inhibition of *α*-amylase than the aqueous extract. A correlation was observed between the IC_50_ values and TPC. The ethanolic extract (20.8 μg GAE/mL), which contained the highest amounts of rutin (110.4 μg/g fw), myricetin (16.6 μg/g fw) and *trans*-resveratrol (13.0 μg/g fw), showed the best inhibitory effect. A correlation was found between the amount of myricetin in the extract and the IC_50_ values, with the efficiency varying in the following sequence of extracts: ethanolic > acetone > methanolic > aqueous.

The synthetic enzyme inhibitors cause gastrointestinal side effects such as diarrhea, abdominal pain and flatulence [33]. The natural metabolites reported from plants have become more valuable targets for discovering treatments for various health disorders, including diabetes [32]. The inhibitory effects of plant polyphenols for *α*-amylase have attracted interest among researchers. Several molecules have been reported to possess *α*-amylase inhibitory activity [33,34,35,36,37].

Güder et al. [38] reported IC_50_ values of the *V. myrtillus* L. extracts in the range from 61.38 to 281.53 μg/mL against *α*-amylase, and compared them with standard acarbose (IC_50_ value of 87.55 μg/mL).

### 2.6. Antioxidant Activity

Along with disturbances in insulin secretion and peripheral insulin resistance, other metabolic processes, including oxidative stress, glucotoxicity, lipotoxicity and low-grade inflammation, develop in the pre-diabetic state [39]. By improving glycemic control and reducing oxidative stress, bilberries as a functional food positively influence diverse pathophysiological mechanisms that occur in this prediabetic state, and for which oral antidiabetic agents are not approved in practice or are only considered in high-risk cases [39].

**Figure 4:**
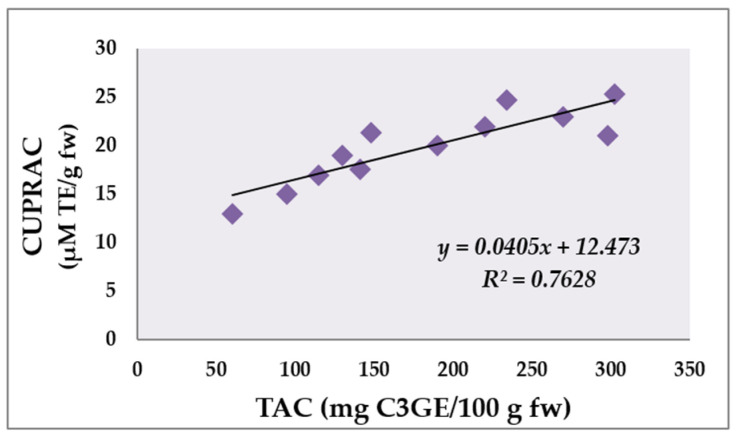
Correlation between total anthocyanin content (mg C3GE/100 g fw) and antioxidant activity measured by CUPRAC assay (μM TE/g fw) of analyzed bilberry extracts.

Phenolic compounds are well known to have antioxidant activity [40,41,42,43,44,45]. The antioxidant capacity of the studied four bilberry extracts was measured by FRAP, CUPRAC and DPPH methods. Experiments with the positive control 125 µM BHT were carried out for comparative purposes (Table 3). The range of FRAP values in the present study from 35.9 to 197.4 μM TE/g fw was generally higher than the value reported by Jurca et al. [46]—73.16 μM TE/g dw. The CUPRAC values for the bilberry fruits varied from 17.6 to 25.3 μM TE/g fw. Other authors reported higher values for blueberry cultivars ranging between 134.76 and 185.78 μM TE/g fw [17]. According to the CUPRAC test, the ethanolic extract possessed the highest activity, followed by the methanolic, acetone and aqueous extracts. As a result of the evaluation of the studies, it was observed that there was a direct correlation between the anthocyanin content of the extracts and the antioxidant capacity values of the CUPRAC method (Figure 4). 

Figure 4 illustrates the correlation between the total anthocyanin content and CUPRAC analysis of plant extracts. The results demonstrated a positive correlation coefficient (r = 0.7628) which was highly significant (*p* < 0.01). In this investigation, it seems that the higher total anthocyanin content of the plant extracts resulted in higher antioxidant activity with respect to the CUPRAC assay.

The DPPH scavenging activity of bilberry extracts varied between 216.5 μM TE/g fw and 376.8 μM TE/g fw. When the radical scavenging capacity of the extracts was considered, the acetone and water extracts showed better activity compared to the ethanolic and methanolic extracts. No significant correlation was observed between the ability to scavenge DPPH radicals and phenolic content. However, the antioxidant capacity of the studied extracts was less effective than that of BHT for the FRAP and CUPRAC tests, while they were found to be more effective for the radical scavenging assay.

## 3. Materials and Methods

### 3.1. Plant Material

The fruits of *Vaccinium myrtillus* L. (*Myrtilli fructus*) were collected from the following Bulgarian floristic region: the Rhodope Mountains of Bulgaria–Yundola area (42.0630° N, 23.8546° E) at 1400 m altitude. The fruits were gathered after full ripening from natural habitats in August and September during the 2022 vegetative season. The species was identified, and the herbarium specimen of the species is stored in the Department of Pharmacognosy and Pharmaceutical Chemistry, Faculty of Pharmacy, Medical University of Plovdiv. The collected fruits were cleaned of impurities, such as other plants, leaves and stem parts, and examined for whether they were damaged, altered, immature or overripe fruits, before being frozen and stored at −79 °C (Lexicon^®^ II Ultra-low Temperature Freezer, Esco Lifesciences Group, Republic of Singapore, Malay Peninsula) until analysis.

### 3.2. Chemicals and Reagents

*Alpha*-amylase (EC 3.2.1.1)—77 U/mg from human saliva type XIII-A, rutine hydrate, quercetin, kaempferol, *trans*-resveratrol, DPPH (2,2-Diphenyl-1-picrylhydrazyl), trolox (6-hydroxy-2,5,7,8-tetramethylchromane-2-carboxylic acid), neocuproine, ammonium acetate, Copper (II) chloride dehydrate, 2,4,6-tris (2-pyridyl)-s-triazine (TPTZ), iron(III) chloride hexahydrate and 3,5-di-tert-4-butylhydroxytoluene (BHT) were purchased from Sigma-Aldrich Chemie (Schnelldorf, Germany). Delphinidin-3-*O*-galactoside chloride for HPLC, Cat. No. 0905, delphinidin-3-O-glucoside chloride for HPLC, Cat. No. 0938 S, malvidin-3-*O*-glucoside chloride for HPLC, Cat. No. 0911 S, cyanidin-3-*O*-glucoside chloride for HPLC and Cat. No. 0915 S were purchased from Extrasynthese (Lyon, France). HPLC grade methanol, acetonitrile and formic acid were obtained from Merck (Darmstadt, Germany). Starch soluble GR (500 gm), 3,5-dinitrosalicylic acid, 2-[4-(2-Hydroxyethyl)-1-piperazinyl]-ethanesulfonic acid buffer substance HEPES, Folin–Ciocalteu’s phenol reagent, formic acid, gallic acid, potassium chloride and sodium acetate were purchased from Merck (Darmstadt, Germany). 

### 3.3. Preparation of Vaccinium myrtillus L. Extracts

#### 3.3.1. Water Extraction

The whole frozen fruits were blended with distilled water—liquid/solid ratio of 20:1 (mL/g). The phenolic compounds from the berries were extracted by using an ultrasonic bath at a temperature of 35 °C for 20 min (Bandelin Sonorex, Berlin, Germany), and filtered through nylon cloth. This process was repeated twice under the same conditions. Both extracts obtained were combined and concentrated to dryness using a Buchi/R-100 rotary evaporator at a temperature not exceeding 50 °C. Then, the dry extract was dissolved in a 15 mL volumetric flask containing distilled water, and adjusted to the volume with water.

#### 3.3.2. Organic Solvent Extraction

The organic solvents (methanol, ethanol and acetone, respectively) and water: formic acid (70/30/1 *v*/*v*/*v*) mixtures were used as the extraction solvent mixture. The ultrasonic bath extraction conditions and preparation of the stock solution were applied as used in the water extraction section above. 

### 3.4. Total Phenolic Content (TPC) Assay

The Folin-Ciocalteu method was used to determine the total phenolic content, as described by Singleton and Rossi [47]. The absorbance readings were taken at 760 nm using the UV–VIS Biochrom Libra S80PC Double Beam Spectrophotometer after incubation for 5 min at 50 °C. Gallic acid was used as the reference standard. The results were expressed as milligram gallic acid equivalent per 100 g of fresh fruits (GAE/100 g fw).

### 3.5. Total Anthocyanin Content (TAC) Assay

The total anthocyanin content of the extracts was determined according to the pH differential method [48], and based on the structural transformations of anthocyanins as a function of the pH generating colored solutions. The absorbance values were measured with a UV–VIS Biochrom Libra S80PC spectrophotometer at 520 and 700 nm. The results were expressed as mg cyanidin-3-glucoside equivalents/100 g fresh fruits (C3GE/100 g fw) using a molar extinction coefficient of 29 600.

### 3.6. High-Performance Liquid Chromatography (HPLC) Analysis of Flavonols and trans-Resveratrol

The flavonol composition and *trans*-resveratrol content of the extracts were assessed by HPLC analysis using the chromatographic system VWR La Prep Σ (Knawer, Germany), which consists of an LP 1100 HPLC pump, LP 3104 UV absorbance detector and column Chromolith^®^ Performance RP-18e (100 × 4.6 mm × 2 μm), sourced from Merck, Darmstadt, Germany. The managerial chromatography system and data processing used EZChrome Elite, a software of Agilent—version 3.2.0. The column temperature was maintained at 25 °C. Mobile phase A was methanol, mobile phase B was acetonitrile and mobile phase C was water. The water used for the analysis had ultrapure-grade water. The mobile phase solvents underwent filtration through 0.45 μm membrane filters (Millipore, Milford, MA, USA) before analysis. The HPLC analysis was performed using an isocratic program as follows: 0–15 min, 46% A to 12% B to 42% C. The injection volume was 20 μL, the mobile phase flow rate was 0.78 mL/min and the detection wavelength was 360 nm for flavonols and 310 nm for *trans*-resveratrol. The samples were determined by the retention time of rutin, myricetin, quercetin, kaempferol and *trans*-resveratrol standards.

### 3.7. High-Performance Liquid Chromatography (HPLC) Analysis of Anthocyanins

Anthocyanins were identified and quantified using the chromatography system from Section 3.6. The program used was isocratic: A/B—60/40 (*v*/*v*), with mobile phase A—formic acid:water in a ratio of 10:90 (*v*/*v*); mobile phase B—formic acid:methanol:water in a ratio of 10:40:50 (*v*/*v*/*v*); column temperature: 25 °C; mobile phase flow rate: 0.7 mL/min; analytical wavelength: 520 nm. The identification of the peaks was conducted by the retention times towards the standards of anthocyanins: delphinidin-3-galactoside, delphinidin-3-glucoside, cyanidin-3-glucoside and malvidin-3-glucoside. The methanol and formic acid (purity: 98–100%) used for this analysis had HPLC grade. The water was of an ultrapure-grade. The mobile phase solvents underwent filtration through 0.45 μm membrane filters (Millipore, Milford, MA, USA) before analysis.

### 3.8. Alpha-Amylase Inhibition Assay

In vitro *α*-amylase inhibition was studied by a previously developed method [33] with modifications. The assay mixture contained 400 μL of substrate (starch solution), 50 μL of inhibitor (extract) of different concentrations and 50 mM of HEPES buffer solution (pH 6.9) containing 50 mM of sodium chloride. The 1% starch solution was prepared in a 50 mM HEPES buffer at 90 °C in a water bath. The enzyme solution (2.57 U/mL) was prepared in the HEPES buffer. The assay mixture was pre-incubated at 37 °C in a water bath for 10 min. The reaction was started by adding the enzyme to the assay solution. After incubation for 10 min at 37 °C, the reaction was stopped by adding 500 μL of the colour reagent (3,5-dinitrosalicylic acid solution) and placing the samples in a water bath at 100 °C for 10 min. Subsequently, the samples were cooled to room temperature and diluted after adding 5 mL of distilled water.

The test samples were accompanied by blank samples containing all the components except for the enzyme to avoid the possible absorbance of the berry extracts. The absorbance was recorded at 540 nm using a Biochrom Libra S80PC spectrophotometer. The inhibition activities on *α*-amylase were expressed as IC_50_ (the concentration required to inhibit *α*-amylase activity by 50%). The IC_50_ values were determined by linear regression analysis using different concentrations in triplicate.

### 3.9. Antioxidant Activity Assay

The antioxidant activities of the samples were determined by using FRAP, CUPRAC and DPPH methods.

The FRAP method was used for the determination of the total antioxidant capacity, based on the reduction of the yellow [Fe^3+^-(TPTZ)2] complex to the blue [Fe^2+^-TPTZ] complex by electron-donating substances under acidic conditions [49]. The 2850 μL of FRAP reagent (containing TPTZ, FeCl_3_ and acetate buffer) and 150 μL of the extracts were allowed to react. Maximum absorbance values at 593 nm were recorded for 10 min at 37 °C.

The CUPRAC assay was performed as described by Apak et al. [50]. The method involves mixing the solutions of 10 mM of CuCl_2_, 7.5 mM of neocuproine, 1 M of ammonium acetate at a pH of 7 and 50 μL of different extracts, and measuring the absorbance at 450 nm after 60 min.

The radical scavenging activity (RSA) of the extracts against 2,2-diphenyl-1-picrylhydrazyl (DPPH) radical was assessed spectrophotometrically at 517 nm. The assay was completed according to a method reported by Bunea et al. [17]. A DPPH solution (80 μM) was freshly prepared in methanol. A volume of 2 mL of this solution was allowed to react with 150 μL of the sample extracts in various concentrations, and the absorbance was measured after 15 min in the dark.

The final absorbance was compared with the standard curve in the range from 25 to 500 μM Trolox, dissolved in methanol. The data were expressed as μM Trolox equivalent/g fresh fruits (μM TE/g fw).

### 3.10. Statistical Analysis

The data obtained in the current study were performed in triplicate (*n* = 3) and expressed as mean values ± standard deviation (SD). Using the SPSS statistical software package (24th version), the data were analyzed via one-way analysis of variance (ANOVA), followed by Tukey’s multiple comparison test at *p* ˂ 0.05 (*p*-values of less than 0.05 were considered statistically significant).

## 4. Conclusions

Our in vitro results established a relationship between the amount of the flavonol myricetin and the degree of the inhibition of *α*-amylase activity. In addition, our results confirm that the polyphenols found in bilberry extracts inhibit *α*-amylase and have the capability to act as antioxidants and free radical scavengers. According to the CUPRAC method, the highest antioxidant capacity was determined for the ethanolic extract of *V. myrtillus* L., and the activity of the extracts was found to be correlated to their anthocyanin content. The highest flavonol glycoside content in bilberries was represented by quercetin-rutinoside (rutin) for the ethanolic extract. Furthermore, the ethanolic extract was the most effective against *α*-amylase. The inhibitory properties of the extracts of *V. myrtillus* L. would serve as a good prerequisite for their successful future application in the suppression of postprandial hyperglycemia in diabetic patients.

## Figures and Tables

**Table 1:** Flavonol and *trans*-resveratrol content in bilberry extracts determined by HPLC analysis.

Extracts	Rutin (μg/g fw)	Myricetin (μg/g fw)	Quercetin (μg/g fw)	Kaempferol (μg/g fw)	Resveratrol (μg/g fw)
Aqueous	100.3 ± 2.9	10.6 ± 0.4	0.7 ± 0.01	2.6 ± 0.1	12.1 ± 0.4
Acetone	95.6 ± 2.7	15.6 ± 0.5	1.7 ± 0.05	ND	11.3 ± 0.4
Ethanolic	110.4 ± 3.8	16.6 ± 0.5	0.6 ± 0.01	ND	13.0 ± 0.5
Methanolic	104.2 ± 3.4	13.5 ± 0.4	1.6 ± 0.06	1.4 ± 0.04	10.6 ± 0.3

The data expressed as mean ± SD, n = 3. ND—not detected.

**Table 2:** Anthocyanin content in bilberry extracts determined by HPLC analysis.

*Vaccinium myrtillus* L. Extracts	Delphinidin- 3-Galactoside (mg/100 g fw)	Delphinidin- 3-Glucoside (mg/100 g fw)	Cyanidin- 3-Glucoside (mg/100 g fw)	Malvidin- 3-Glucoside (mg/100 g fw)
Aqueous	38.3 ± 1.9	16.2 ± 0.8	18.7 ± 0.9	27.4 ± 1.3
Methanolic	79.4 ± 3.8	24.2 ± 1.2	19.2 ± 0.9	37.8 ± 1.9

The data expressed as mean ± SD, n = 3.

**Table 3:** Antioxidant activity of analyzed bilberry extracts measured by FRAP, CUPRAC and DPPH assays.

	FRAP (μM TE/g fw)	CUPRAC (μM TE/g fw)	DPPH Scavenging (μM TE/g fw)
***Vaccinium myrtillus*** **L. extracts**			
Aqueous	35.9 ± 1.62	17.6 ± 0.68	298.1 ± 11.92
Acetone	197.4 ± 8.9	21.4 ± 0.73	376.8 ± 18.84
Ethanolic	57.3 ± 2.31	25.3 ± 1.02	271.4 ± 16.28
Methanolic	39.0 ± 1.68	24.7 ± 1.01	216.5 ± 10.83
**Positive control**			
BHT 125 μM	153.2 ± 6.13 μM TE	157.9 ± 7.89 μM TE	354.4 ± 14.18 μM TE

The data expressed as mean ± SD, n = 3.

## Data Availability

Not applicable.
